# The bacterial signature offers vision into the machinery of coral fitness across high‐latitude coral reef in the South China Sea

**DOI:** 10.1111/1758-2229.13119

**Published:** 2022-08-31

**Authors:** Hala F. Mohamed, Amro Abd‐Elgawad, Rongshuo Cai, Zhaohe Luo, Changan Xu

**Affiliations:** ^1^ Third Institute of Oceanography Ministry of Natural Resources Xiamen People's Republic of China; ^2^ Al‐Azhar University (Girls Branch) Faculty of Science, Botany & Microbiology Department Cairo Egypt; ^3^ Tourism Developing Authority Central Adminstration for Environmental Affairs Cairo Egypt

## Abstract

Coral–bacterial interaction is a major driver in coral acclimatization to the stressful environment. 16S rRNA High‐throughput sequencing was used to classify the role of different coral reef compartments; sediment, water, and tissue; in the South China Sea (SCS), as well as different locations in shaping the microbial community. The majority of OTUs significantly shifted at impacted sites and indicated distinction in the relative abundance of bacteria compartment/site‐wise. Richness and diversity were higher, and more taxa were enriched in the sediment communities. Proteobacteria dominated sediment samples, while Cyanobacteria dominated water samples. Coral tissue showed a shift among different sites with Proteobacteria remaining the dominant Phylum. Moreover, we report a dominance of *Chlorobium* genus in the healthy coral tissue sample collected from the severely damaged Site B, suggesting a contribution to tolerance and adaptation to the disturbing environment. Thus, revealing the complex functionally diverse microbial patterns associated with biotic and abiotic disturbed coral reefs will deliver understanding of the symbiotic connections and competitive benefit inside the hosts niche and can reveal a measurable footprint of the environmental impacts on coral ecosystems. We hence, urge scientists to draw more attention towards using coral microbiome as a self‐sustaining tool in coral restoration.

## INTRODUCTION

The South China Sea (SCS) represents the north side of the ‘Coral Triangle’. Many fringing reefs and coral population are scattered on the north sideway of the SCS and their dispersal is attributed to the subtropical climate (Chen et al., 2019). Tropical coral reef is considered as one of the greatest significant biodiverse communities worldwide, which supports human through a variety of aspects such as livelihood, fisheries, erosion prevention, tourism, and defence against distressed ecological changes (Polidoro & Carpenter, 2013). Thus, facing a tremendous decline caused by anthropogenic disturbances and climate change, which led to coral diseases and bleaching events. Scientists have been actively trying different approaches for coral restoration in order to save corals from further decline (Abd‐Elgawad et al., 2022; Feng et al., 2022; Yao et al., 2020). Water column, sediment as well as coral tissue, skeleton and mucus are different compartments harbouring endosymbiotic microbiome including bacteria, microalga, viruses, archaea and fungi, (Osman et al., 2020). As corals have an active relationship with these various associates, (Brener‐Raffalli et al., 2018), corals acclimatize to broad environmental pressures by rearrangement of their microbiome (specifically the assembly of the bacteria and Symbiodiniaceae) (Ziegler et al., 2019). Understanding the community composition of symbiotic bacteria and its fluctuations is exclusively significant to understand the acclimatization of corals to diverse ecological conditions and their tolerance to climate change, which in consequence, support the hypothesis of using coral microbiome for coral restoration (Mohamed et al., 2022). Biotic and abiotic features have shown to influence the assembly of bacterial communities in corals (Garren & Azam, 2012; Mouchka et al., 2010; Sharp & Ritchie, 2012). Since Weizhou Island is a high‐latitude area, it was hypothesized that it can benefit from climate change and temperature rise; however, corals in the area are continuing to decline suggesting the side factors affecting coral growth.

Many studies reported that coral decline is closely associated with human activity caused water eutrophication (D'Angelo & Wiedenmann, 2014; Lesser, 2021; Li et al., 2017; Szmant, 2002; Zhu et al., 2021), and this might be the case in Weizhou Island, which explains their contradictory to the refugee theory in high‐latitude areas. This suggestion could be found by Shi et al. (2010) who pointed out that the main factor of coral degradation in Hainan fringing reef is water eutrophication caused by sewage and waste discharge. Moreover, Yu et al. and Zhoe et al. further reported that low coral cover in SCS is more associated with human activity rather than bleaching caused by global warming (Yu et al., 2019; Zhao et al., 2014).

Several studies have reported that pH, nutrients such as; nitrogen, and phosphorus; temperature, salinity, dissolved organic carbon and physical structure of substrate, affect coral health especially those with calcium carbonate skeleton and their associated microbiome (Hoegh‐Guldberg et al., 2017; Roik et al., 2016; Yu et al., 2019). Biological measures, such as diseases, algal overgrowth and, changes in the environmental parameters cause alterations in the richness, structure, and dominance of coral‐symbiotic bacteria (Ceh et al., 2012; Cróquer et al., 2013; Littman et al., 2011; Morrow et al., 2015; Yu et al., 2021).

Thus, coral‐symbiotic bacteria are considered as an effective sign of coral fitness (Thompson et al., 2015; Zhu et al., 2021) as they play substantial function in sustaining the healthiness of coral reef networks, biogeochemical cycles and nutrient transformation (Yu et al., 2021). Many bacterial groups can assist coral acclimatize to rise in sea surface temperature (SST) (Osman et al., 2020), eutrophication, and turbidness (Ziegler et al., 2017). For example, heterotrophic *Synechoccus* and Pickoplankton show more richness in the less saline and warmer waters. Shift of the coral microbiome of *Fungia granulosa* as a reaction to high salinity has been reported (Röthig et al., 2017), which explains the developed resistance of this species to high salinity environment in the Red Sea. Also, Roder et al. (2015) revealed that highly arranged microbiomes of *Ctenactis echinata* were spotted at locations where *C. echinata* was most dominant; these locations were described as rocky substrates and pure water, suggesting that microbiome structure propose environment competence.

Apart from climatic disturbances, human impact also has a dramatic effect on the general coral health and its associated microbiome worldwide such as ocean acidification, elevation of inorganic nutrition level, introduction of organic compounds through sewage, and sedimentation. Land use and fishing are also associated with rearrangements of the microbial multiplicity (Lamb et al., 2015; Mohamed et al., 2022). Also some studies reported that pH decline from 8.2 to 7.3 stimulated the growth of the pathogenic bacteria Vibrionaceae and Alteromonadaceae in *Acropora eurystoma* coral but not Gammaproteobacteria nor Cyanobacteria (Muscatine et al., 1989). In addition, many bacteria exhibited an increase in richness as a response to poor water quality as a result of high nutrient concentrations, like *Acropora hemprichii* in contrast to others, such as Endozoicomonas, which usually exhibit no obvious alteration (Neave et al., 2017). Also, microbial symbionts in coral mucus exhibited some interruption (Al‐Farawati et al., 2011) in response to increased organic carbon, which supported other microorganisms of no favour to coral fitness. Many studies reported host specificity of coral microbiome in healthy corals (Sunagawa et al., 2010) and disease specificity in diseased corals regardless of the species or the geographical region (Roder et al., 2015). For example, Vibreonacea and Rhodobacteraceae taxa are frequently linked to coral bleaching and diseases (Rosenberg & Falkovitz, 2004). Abundance of such species leads to change in the functioning of the normal associated microbial community and hence causing disease (Thurber et al., 2009). Sometimes, a combination of anthropogenic impact and climate change is responsible for the microbial community shift and the development of coral diseases, bleaching and decline.

**FIGURE 1:**
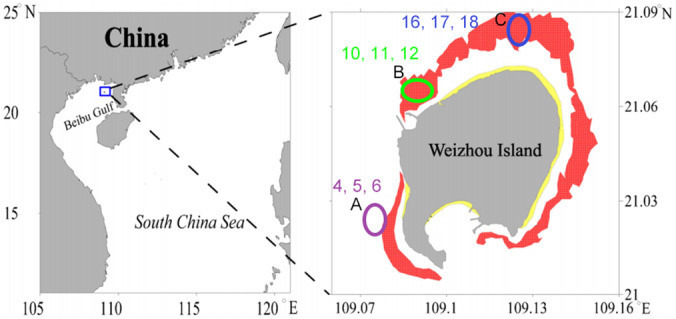
Map for Weizhou Island. On the left, drawing map for Weizhou Island position, in the middle, a sat image illustrating the 21‐study stations, and on the right, the chosen nine sampling stations. *Source*: The red zones are living coral cover areas modified from the studies by Huang et al. (2019) and Ning et al. (2019). The unfilled circles represent sampling positions A, B and C. Purple circle, represents the moderately impacted Site A, with three stations covered (4,5,6).

Many research studies investigated the local human activity impact on coral reefs in Weizhou Island, the biggest and youngest volcanic island in China Sea, in a trial to understand why it could not benefit from the climate change and the SST rise according to the refuge theory (Wang et al., 2020). However, the microbial community in Weizhou Island coral reefs remains unexplored especially in correlation with the two types of impact. The coral ecological success and tolerance rely on the associated symbionts including bacterial community in the surrounding water and sediment substrates, which are shaped according to the water quality that reflects anthropogenic activity impact as well as global change (Zhang et al., 2016). Here, we examined the bacterial community composition in water column, sediments, and coral tissues at three coral reef sites in Weizhou Island (Figure 1) that were designated to be heavily exposed to anthropogenic and climatic disturbances, relatively unimpacted, and nonimpacted healthy reef according to their coral cover (living and dead), which made them model areas to study the present bacterial community in relation to the coral health using high‐throughput sequencing and network analysis to improve the vision into bacterial assembly related to Weizhou Island coral reefs. We were attentive to illuminate the difference of bacterial community structure and abundance among various coral compartments as well as different sites with different coral health, in addition to discovery of bacterial input to functional acclimatization to the distinct environment in northern SCS. Our results are in consistence with former studies that have revealed that coral‐bacterial symbionts (holobionts) are very much dependent on the environmental surroundings that can vary among distances of several kilometres (between reefs and locations). Studies on coral reef bacterial communities are significant for explaining the health status of corals (Littman et al., 2010) in a specific location because they indicate the particular category of bacteria exist through the sampling time (indicative of contaminated spots or climatic disturbed sites). A specific change in the dominance and rise in the abundance of certain genus, species or families of a bacterial assembly can reflect an inadequate presence that should be assessed and managed by reef administrators. We conducted a comparative analysis of the microbial structure of three coral reef compartments; sediment, water and tissue from the three chosen sites A, B and C. Tissue samples were randomly collected from the coral colonies from each of sites A and B, which are exposed to two types of influences, local sewage and sedimentation but with different levels and were compared with corresponding samples from slightly impacted Site C.

## EXPERIMENTAL PROCEDURES

### Study site, sampling stations and environmental stresses

The area selected for performing the current study is located 21 km south of Beihai city, the biggest island in Guangxi and one of the most beautiful islands in China, which extends in Beibu Gulf (Figure 1). The island area is impacted with different climatic disturbances as well as anthropogenic activities, and developments (Ning et al., 2019), such as water warming, and rainfall (Chen et al., 2021; Feng et al., 2022), pollution from petroleum companies, and shipping especially Site B in addition to touristic impact. In addition, overfishing in the reef area is likely to reduce the grazing animals which feed on benthic algae, thus allows for algal expansion over the corals and compete for space, hence, affect also their bacterial associated communities (Morrow et al., 2013). Moreover, the region also suffers from frequent cyclones, which led to increased sedimentation and turbidity. These have caused a sever loss of coral communities and associated habitats. The combination of these influences has led to diminishing of hard coral cover in the zone (Yu et al., 2019).

The core substrates in Weizhou Island are gravel, reef patch, rock and sand (Wang & Wang, 2016). Gravel and sand are heavily dispersed in the north side, whereas rocks are present in the south‐east of Weizhou Island. Three coral reef sites, A, B, and C covering 21 stations around this area (between 21°1.246′ N, 109°4.632′ E and 21°5.008′ N, 109°7.488′ E) (Figure 1) were selected using Global Positioning System (GPS) for screening the benthic substrate scheme and coral coverage in response to latitude and climate, line intercept transect techniques were used, as described by the Australian Institute of Marine Science and recommended by the Global Coral Reef Monitoring Network (English et al., 1997; Hill & Wilkinson, 2004; Zhao et al., 2013; Zhao et al., 2016). Three replicate line transects were laid parallel to the shoreline at two depths (3.5 and 7.5 m) representing the shallow and deep zones of the reef front. The averages of the three replicates were calculated to represent the percentage value for each site.

Nine of these stations (4, 5, 6, 10, 11, 12, 16, 17, 18) were chosen as replicates for performing microbial community analysis of sediment, water, and coral tissues in relation to coral health status using high‐throughput sequencing analysis. All sites were classified as sandy reefs with some patches of *Pavona* corals, especially Site C. Data for each site location including location names, coordinates, and area description are shown in Table 1. All samples were collected on June 2020 using the Academic Reefs Systems Unit (RSU) boat. Sites B, A and C represent the severely, moderately, and slightly damaged coral reef, respectively.

**TABLE 1:** Surveyed site description indicating station position, nature of coral reef, coral coverage (living and dead corals), non‐coral coverage (rock, sand, and coral rubbles)

Site	Station no.	N	E	Coral reef structure	Coral coverage (%)		Non‐coral coverage (%)
					Dead coral (%)	Living coral (%)	
1	1	21°2.6849′ N	109°8.7512′ E	Sandy reef	7.7	16.3	76
	2	21°2.801′ N	109°8.729′ E				
	3	21°2.563′ N	109°8.778′ E				
2(A)	4	21°1.3966′ N	109°4.6977′ E	Sandy reef with *Pavona* patches	10.3	42.7	47
	5	21°1.635′ N	109°4.807′ E				
	6	21° 1.246′ N	109°4.632′ E				
3	7	21°1.2087′ N	109°5.7115′ E	Sandy reef	6	7	87
	8	21°1.256′ N	109°5.655′ E				
	9	21°1.137′ N	109°5.685′ E				
4(B)	10	21°3.9070′ N	109°5.5701′ E	Sandy reef with coral rubbles	3.7	3.3	93
	11	21°3.978′ N	109°5.748′ E				
	12	21°3.910′ N	109°5.440′ E				
5	13	21°4.8833′ N	109°6.6117′ E	*Pavona* patches and coral rubbles	10.7	34.3	55
	14	21°4.792′ N	109°6.519′ E				
	15	21°4.859′ N	109°6.704′ E				
6(C)	16	21°5.0074′ N	109°7.5813′ E	Sandy reef with *Pavona* patches	25.5	54.5	20
	17	21°4.916′ N	109°7.668′ E				
	18	21°5.008′ N	109°7.488′ E				
7	19	21°4.9918′ N	109°6.5311′ E	Sandy reef	5.3	32.8	62
	20	21°5.014′ N	109°6.819′ E				
	21	21°4.918′ N	109°6.411′ E				

*Note*: Seven sites are shown with 21 stations. Sites A, B and C represent the moderately, severely and slightly damaged coral reefs.

### Sample collection

#### Coral and water sampling

In the severely damaged Site B, only one surviving visually healthy‐looking adult coral colony of *Porites lutea* was spotted and no more visibly healthy colonies were detected. This strengthen the heavy impact on this site and support the hypothesis that this escaping coral colony had a microbial pattern that might be associated with acclimatization and resilience which drawn our attention to sample it for analysis of the microbial assemblage. Coral tissue was sampled (Bht) in triplicates at a 5 m depth, by removing few branches to examine the associated bacterial structure for coral resilience. Nine Samples of the same species *Porites lutea* were randomly collected in triplicates for coral colonies from three coral communities A, B and C in Weizhou Island, for best comparison with the healthy colony Bht. (Table 1). Triplicates of the same sample were mixed in one tube. Coral fragments (~3 cm^2^) were sampled using chisel and hammer from a depth of ~5 m through SCUBA diving. The specimens were washed using sterile seawater to guarantee they are free of contamination with living bacteria. All fragments were transferred immediately in pre‐loaded 25 ml Falcon tubes containing 20% dimethyl sulphoxide (DMSO) buffer, then immediately stored at −20°C until used later for DNA extraction.

Nine water samples (each 1 L) were collected in triplicates at the equivalent depth as the coral tissue samples, transferred in insulated cooler and were filtered using 0.22 μm Durapore PVDF filters (Millipore, Billerica, MA, USA) which were then kept at −20°C until transported to the laboratory.

#### Sediment sampling

Nine samples were collected in triplicates from coral reef sediment of Sites A, B and C and triplicates of the same sample were mixed in one tube. Three extra sediment samples (Bhs) were collected in triplicates nearby the healthy colony sampled from Site B. Samples were collected in a single line transect with scuba diving equipment using a chisel and a hammer. Three replicate's line transects were placed parallel to the shoreline at two depths (3.5 and 7.5 m) representing the shallow and deep zones of the reef front. Samples were preserved on ice box until transferred to −20°C. The average of the three replicates of each line transects represents the percentage values for each site.

### Water quality measurement in the local environment of Weizhou Island

The actual chemical and physical parameters indicating water quality have been assessed for the three Sites A, B and C in June 2020 (Table S1).

The SST, particulate organic carbon and nutrition concentration, of the three selected sites in Weizhou Island were tested (Table S1). In addition, dissolved oxygen and salinity were measured (Table S1), as crucial environmental variables to impact microbial community structure in corals (Röthig et al., 2016; Zheng et al., 2014). The average annual SST in Weizhou Island is 24.62°C; the average annual air temperature is 22.6°C; the average annual precipitation is 1380.2 mm; mean wind speed is 4.8 m/s (Yu et al., 2019). Former study has described that monthly average salinity at Weizhou Island ranged from 31.17 to 32.66 over 20 years (Yang et al., 2006). The dissolved oxygen is helpful to coral development and reproduction, its saturation is over 90 and no anoxia was detected through the year (Zheng et al., 2014). Weizhou Island is located in a fairly high, subtropical latitude well characterized of rainfall, and ambient sunlight. The pH of the water fluctuates from 8.0 to 8.23. Ocean transparency ranging from 3.0 to 10.0 m; in addition to cyclonic flow all through the year (Yu et al., 2019).

### Microbiome identification and profiling

Coral tissue fragments, sediment and PC filters were all kept at −20°C. Prior to DNA extraction which was performed using a CTAB protocol combined with Zymo DNA Clean & Concentrator kit (Zymo Research Corp, Irvine, USA) following the manufacturer's methodology.

The primers 341F (5′‐CCTACGGGNGGCWGCAG‐3′)/806R (5′‐GGACTACHVGGGTATCTAAT‐3′) (Caporaso, 2010; Muyzer, 1993) targeting the V3–V4 domain of prokaryotic SSU rDNA were used. The amplification protocol in the methods of Ammon (von Ammon et al., 2019) was followed. PCR reaction was performed using The Ex Taq PCR kit (Takara Bio, Japan) according to instructions and primers concentration of 0.2 μM. The PCR cycles involved pre‐denaturation at 95°C for 5 min, 34 cycles of denaturation at 95°C for 30 s, 56°C for 30 s, annealing at 72°C for 30 s and extension at 72°C for 5 min. Amplicons were cleaned using AxyPrep DNA gel extraction kit (Axygen, Union, USA) according to the manufacturer's protocol. Purified PCR products were assessed with an ABI StepOnePlus Real‐Time PCR System (Life Technologies). Sequencing libraries were produced with the NEBNext® Ultra™ DNA Library Prep Kit for Illumina® (New England Biolabs, MA, USA). The 16S rRNA libraries were assessed and sequenced on an Illumina MiSeq platform (Illumina, San Diego, USA) using a paired‐end (2 × 250 bp) HiSeq Reagent Kit following manufacturer's instructions. The software package DADA2 (Divisive Amplicon Denoising Algorithm;Callahan et al., 2016; Callahan et al., 2017) in R was used for analysing sequences and they were quality filtered, merged, dereplicated, and chimeras were removed using the DADA2 workflow (Callahan et al., 2016; Callahan et al., 2017) to detect amplicon sequence variants (ASVs). For 16S sequences, the ASVs were aligned to the SILVA rRNA database (Quast et al., 2013); https://www.arb-silva.de/. Illumina next‐generation DNA sequences were deposited in the sequencing read archive (SRA) of the National Centre for Biotechnology Information (NCBI) under BioProject accession PRJNA851196 ID: 851196, Biosample accessions SAMN29213270‐SAMN29213302.

### Statistics and bioinformatic analysis

The α‐diversity analyses of bacterial community composition were conducted in R environment (R Core Team, 2017) using the package vegan (Oksanen et al., 2020). The α‐diversity, including the ASV richness, ACE (Chao & Yang, 1993), Chao1 (Chao, 1984), Pielou index (Pielou, 1969), Simpson diversity (Simpson, 1949), Shannon diversity (Shannon & Weaver, 1949), rarefaction curves were conducted using R. The venn diagrams were using venny's on‐line website (https://bioinfogp.cnb.csic.es/tools/venny/index.html). The β‐diversity analyses of bacterial Bray–Curtis dissimilarity were performed using the vegdist function in vegan package. Mantel test was run with vegan in R to find link between bacterial community and the environmental parameters (based on Bray–Cutis dissimilarity index).

## RESULTS

Here, we accomplished a comparative analysis of the microbial community composition of coral reefs in different compartments, water, sediment, and coral tissue, from the three selected sites in Weizhou Island according to their observed coral health status. Moreover, microbial community of randomly collected healthy coral tissues found in the severely damaged Site B was examined for mechanism of adaptation to environmental stress.

### Site description

Seven sites in Weizhou Island covered a total of 21 stations have been surveyed and described according to position (Figure 1), nature of coral reef structure, coral coverage (including both dead and living corals), and non‐coral coverage as indications for the coral health status in the studying area (Table 1). Water quality has been also described for the selected sites (Table S1). Out of the seven sites, three have been chosen to carry on with the microbiome analysis in this study, based on their coral health status; Sites 2, 4 and 6 that were renamed as A, B and C, respectively. These three sites were chosen to represent the moderately, severely, and slightly damaged coral reefs, respectively, as shown in Table 1. The three sites suffered from different degrees of natural sedimentation. The highest living coral coverage was obtained in Site C, while the lowest was obtained in Site B and the moderate coral coverage in Site A (Table 1). Sites A, B and C exhibited 53%, 7% and 80% coral cover with 42.7%, 3.3%, and 54.5% living corals, respectively (Table 1) and were chosen to represent the moderately, severely, and slightly damaged coral reefs in this study.

### Sediment and overlying seawater variables

SST, pH, salinity, DO, TSS, NH_4_‐N, NO_2_‐N, COD, PO_4_‐P, SiO_3_‐Si, NO_3_‐N, inorganic N, non‐ionic‐ammonia, oil, Hg, Cu, Pb, and Gr for the selected three sites are shown in Table S1.

In general, the nutrient content in the water of Weizhou Island is quite low; however, Site A showed higher level of DIN compared to the other sites, although Site C showed the highest DIN/DIP ratio. This could be due to the closest contact with the shoreline and being highly susceptible to human impact in the area. The contents of nitrogen (N), phosphorus (P) and silicon (Si) were 0.083–0.1620, 0.005–0.012 and 0.1881–0.2349 mg/L, respectively. The active phosphate (PO_4_‐P) had a potential limiting effect on the reproduction and growth of phytoplankton in the monitoring area. Heavy metal, such as Hg, Cu, Pb, and Gr are in accordance with the First‐class Quality Standard of China (GB 3097‐1997).

### Microbiome assemblages for coral tissue, sediment, and seawater from three sites at high and low taxonomic levels

**FIGURE 2:**
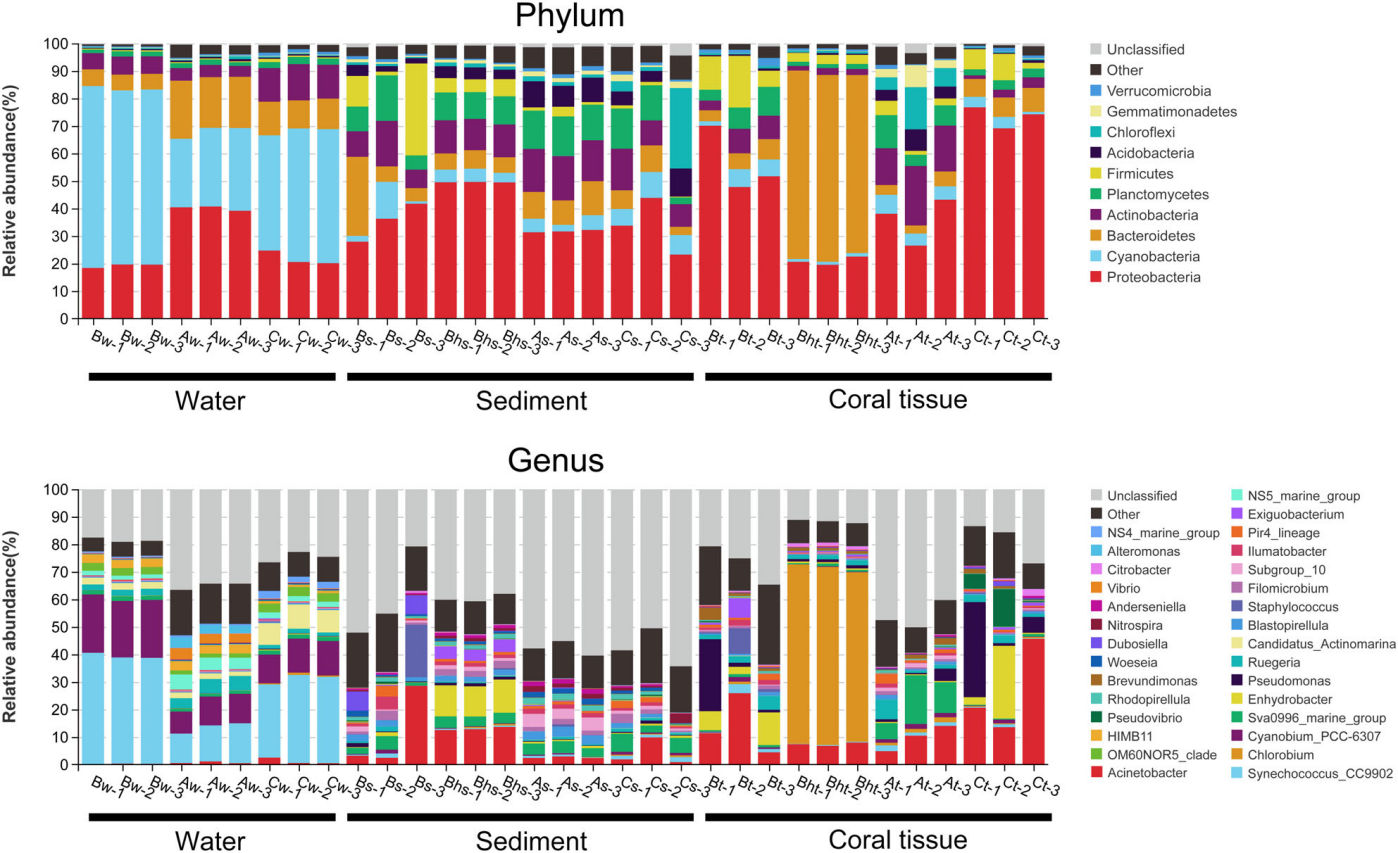
Relative abundance of major 10 bacterial phylum and genus groups based on OTUs derived from the SSU rDNA of different sampling. Each colour denotes one of the 11 most abundant phylum or genus (overall sequence count) in all samples. The rest of other taxa are indicated under group ‘others’. BW, AW, and CW represent water sample from Sites B, A, and C sites, respectively. Bs, Bhs, As, Cs represent sediment samples from B, near healthy colony collected from Sites B, A, and C site, respectively

Sequencing of the 16S rRNA gene amplicon was conducted for a total of 33 samples as follows; three sample replicates from each of sediment, water column and coral tissues collected from the nine stations (4, 5, 6, 10, 11, 12, 16, 17, 18) (Table 1 and Figure 2), in addition to three samples collected from only one unique healthy coral colony observed at the most severely damaged Site B (Bht) and another three samples collected from sediment near to this healthy colony (Bhs) as triplicates to describe microbial community structures for a total of 33 samples. The healthy coral colony isolated from Site B was identified to be (*Porites Lutea*) and the damaged tissue replicates isolated from the same site was also chosen from the same species for best comparison of associated microbiome. Clean 16S tags, a total of 3, 918, 473, and sequence reads with a N90 length of 441 bp were obtained after filtering unqualified sequences (Table S2). The three sets of the sediment, seawater and coral tissue samples, yielded 15,476 unique OTUs (resembling 97% sequence similarity) accounting for 39 bacterial phyla (based on SILVA classification), with a maximum of 4585 unique OUTs in sample As‐1 (Table S2). A total of 36, 34, 33 phyla, and 389, 351, 398 genera were spotted in Sites B, A and C water respectively, a total of 34, 35, 33 phyla, and 377,385,361 genera were distinguished in Sites B, A and C sediment, respectively, and an overall of 33, 30, 30 phyla, and 343, 323, 357 genera were detected in Sites B, A and C coral tissue, respectively.

Other abundant phyla generally detected in all samples in addition to Proteobacteria and Cyanobacteria were Bacteroidetes (8.9% of total sequences), Actinobacteria (9.7%), Planctomycetes (6.4%), Firmicutes (4.0%), Acidobacteria (2.9%), Chloroflexi (2.8%), Gemmatimonadetes (2.4%), Verrucomicrobia (1.0%). The rest of the phyla were detected in lesser numbers, <1% of the whole sequences.

Archaea represented a low abundance percentage (0.04%–1.51%) of combined Bacteria and Archaea. We only focus on the Bacteria in the following analysis. The three technical replicates for each sample displayed similar microbiome assemblages (Figure 2). Microbiome assemblages of sediment, seawater and coral tissue exhibited different patterns among different sites at all taxonomic levels. On the phylum level (Figure 2), Proteobacteria dominated sediment samples, which ranged from 32.1% to 35.5% of the total sediment retrieved sequences, while Cyanobacteria dominated the water samples, ranged from 27.9% to 64.4%, followed by Proteobacteria which ranged from 19.2% to 40.2% (Figure 2). It is worth mentioning that Phylum Firmicutes showed highest abundance with tissue and sediment compartments isolated from Site B compared to the other two sites. Many Firmicutes can form stage of inactive spores that are highly resistant to surrounding disturbances. Those spores carry sufficient energy for germination and are precisely acclimatized to rapidly respond to substrate accessibility and establishment of vegetative cells, which are able to reproduce.

Interestingly, out of all the examined samples, Phylum Bacteroidetes was significantly abundant in one sample (the coral tissue replicates from healthy colony collected from severely impacted Site B, Bht) questioning for a functional contribution to the mechanism of resistance and acclimatization to the disturbed environment.

On the Order level, Pirellulales and Microtrichales were major groups in the sediment of the three sites, with 7.5% ± 1.7% and 7.3% ± 1.0% of total reads. Abundance of Microtrichales in sediments, suggest it as a key heterotroph for carbon mineralization. In addition, Site C showed higher abundance of Pseudomonadales followed by Site B. Synechoccales, Rhodobacterales and Flavobacteriales were major groups of water samples with different relative abundances. In tissue samples, Sites A and B showed similar pattern but with different relative abundance of bacterial groups. Starting with Psuedomonadales as the highest abundance followed by Rhizobiales and Rhodobacterales. While tissue samples from Site B exhibited highest and dominant abundance Microtrichales followed by Psuedomonadales (Figure S1).

On the genus level, *Synechoccus* (Cyanobacteria) was characteristic to water samples followed by *Cyanobium*, *Candidatus_Actinomarina*. *Rugeria* showed higher abundance in Site B compared to Sites A and C (Figures 2 and S1). For sediment samples, Acinetobacter was most abundant followed by Staphylococcus and Sva 0996 marine group, while tissue samples exhibited highest abundance of Acinetobacter in Sites A and C followed by *Enhydrobacter* then *Psuedomonas*. On the other hand, Site B showed high abundance of Acinetobacter followed by Sva099b marine group (Figures 2 and S1). (Bhs) sample from sediment collected near the healthy colony from severely damaged Site B showed relative abundance of *Enhydrobacter* (11.5%) compared to Bs (0.6%) sample collected from sediment near damaged coral tissue from the same site. (Bht) sample collected from tissue of the only healthy colony found in the same site revealed a significant abundance (63.89%) of *Chlorobium* compared to other tissue samples from all sites (Figures 2 and S1), which showed abundance of (0.3%, 1.4%, and 0.4%) from Bt, At and Ct, respectively.

Though typically dominant in soils (Peralta et al., 2013), the abundances of Proteobacteria, Actinobacteria, and Acidobacteria in decomposition‐influenced soils have been formerly described (Carter et al., 2015; Finley et al., 2016) who also described their abundance in decomposition impacted soil.

**FIGURE 3:**
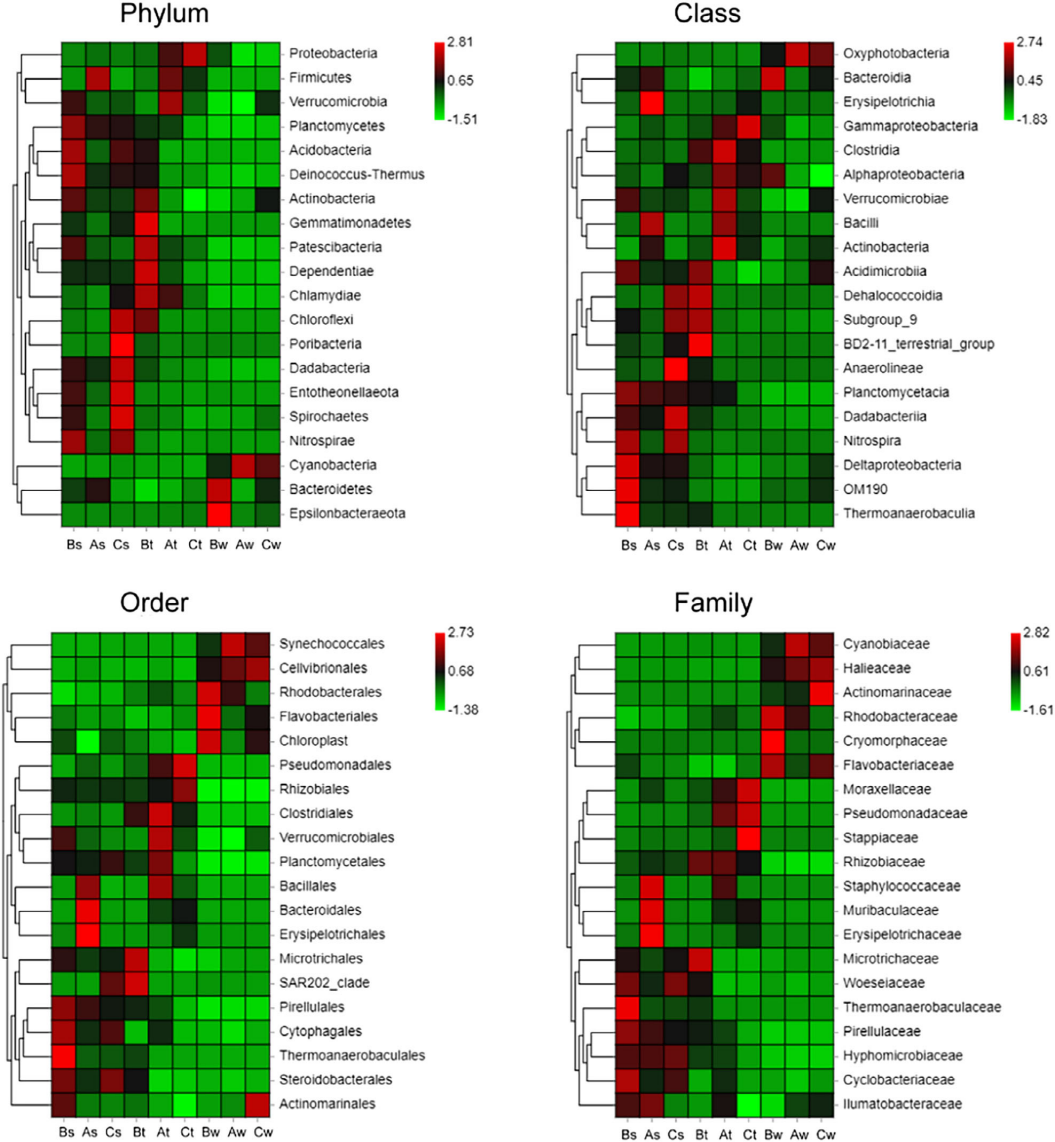
Heat map analysis showing the bacterial group abundance of different sites and different reef compartments (sediment, tissue, and water)

The pictured distinction in community pattern among sediment, seawater and coral tissue samples using heat map of the relative abundance of the 20 most abundant OTUs and a ranked clustering dendrogram displayed average connections among groups (Figure 3).

### Microorganism richness and diversity analysis

As regard to coral reef compartments‐wise, the Alpha diversity index of sediment was higher than the water column and the water was higher than the coral tissue. For different sampling sites, Site A showed the highest Alpha diversity index with all compartments. For the coral tissue, Site A showed the highest Alpha diversity, and Site B showed the lowest alpha diversity. Bht sample showed higher diversity than the diseased sample Bt and Bhs showed higher diversity than Bs (Table 2).

**TABLE 2:** Summary statistics of alpha diversity indices of 16S rRNA‐based bacterial community composition of water, sediment, and coral tissue sample groups

Sample group	Sobs	Shannon	Simpson	Chao1	ACE	Good's coverage	Pielou	PD‐tree
Bw	2143.7	4.6	0.799	3122.6	3397.5	0.982	0.418	280.5
Aw	2678.3	6.9	0.962	3380.5	3693.8	0.981	0.608	340.9
Cw	2618.7	5.9	0.885	3468.3	3724.8	0.981	0.520	326.2
Bs	3390.0	8.2	0.960	3971.6	4151.4	0.982	0.695	393.9
Bhs	3526.3	8.4	0.973	4272.1	4408.2	0.981	0.709	395.9
As	4162.7	9.9	0.997	4759.1	4881.8	0.981	0.826	453.5
Cs	3244.7	8.8	0.990	3870.5	4029.3	0.983	0.751	369.1
Bt	1083.0	6.6	0.952	1458.8	1481.8	0.994	0.665	151.1
Bht	1104.0	3.7	0.588	1668.3	1564.9	0.993	0.365	154.8
At	2176.3	7.8	0.980	2634.9	2649.8	0.989	0.708	264.1
Ct	1453.7	5.6	0.873	2001.8	1978.4	0.991	0.530	201.0

**FIGURE 4:**
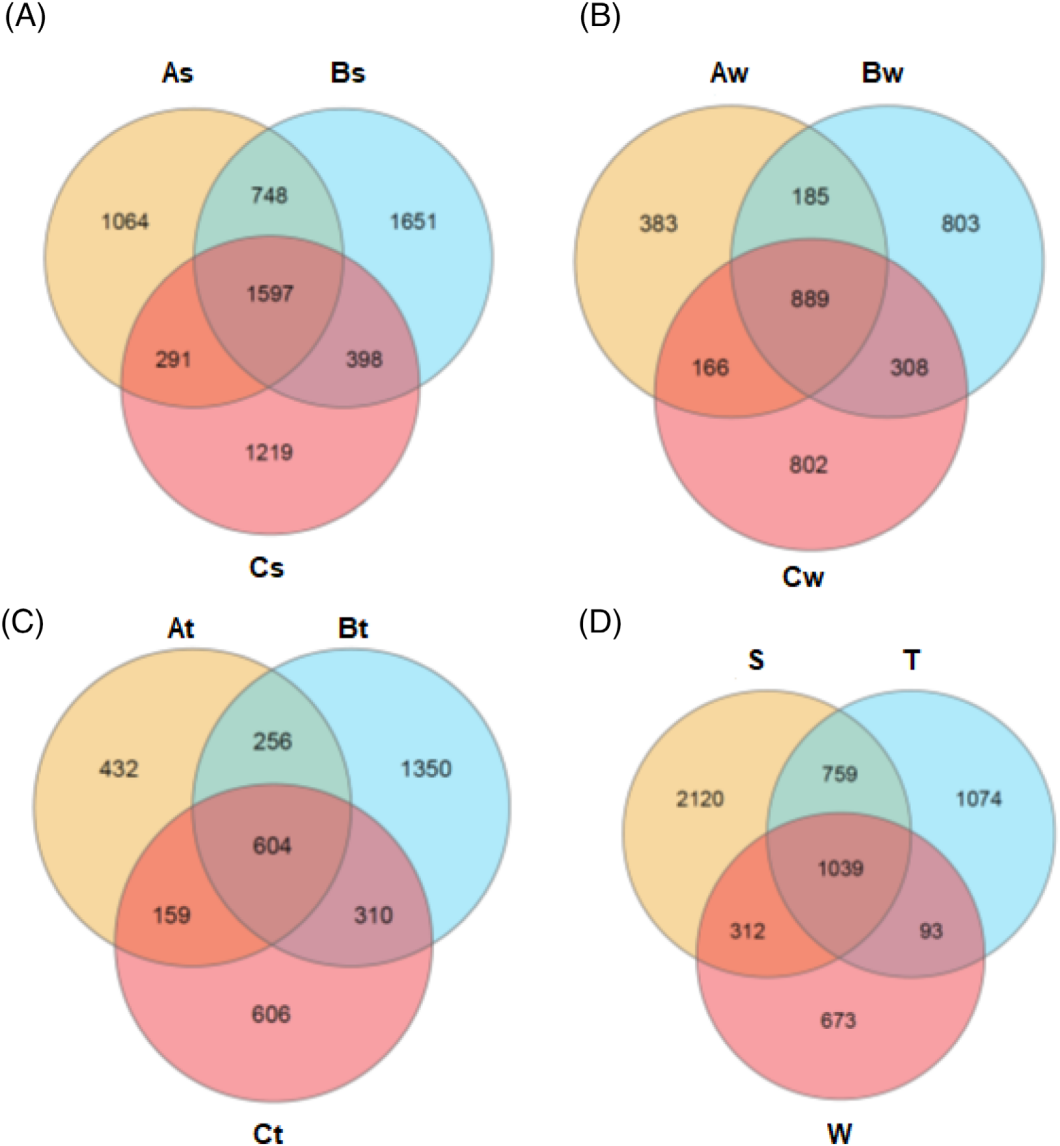
Venn diagrams illustrating the unique and shared microbial OTUs among (A): the sediment compartments from the three sites, (B) the water compartments from the three sites and (C): the tissue compartments from the three sites and, (D): the different reef compartments from the three sites collected together

As shown in Figure 4A–C, 1597, 889, and 604 OTUs were shared among the sediments, water, tissue samples collected from the three sites, respectively. Moreover, each reef compartment was grouped together from the three sites and represented in Figure 4D for comparison of different compartments. Venn diagram indicated 1039 shared OTUS among them.

**FIGURE 5:**
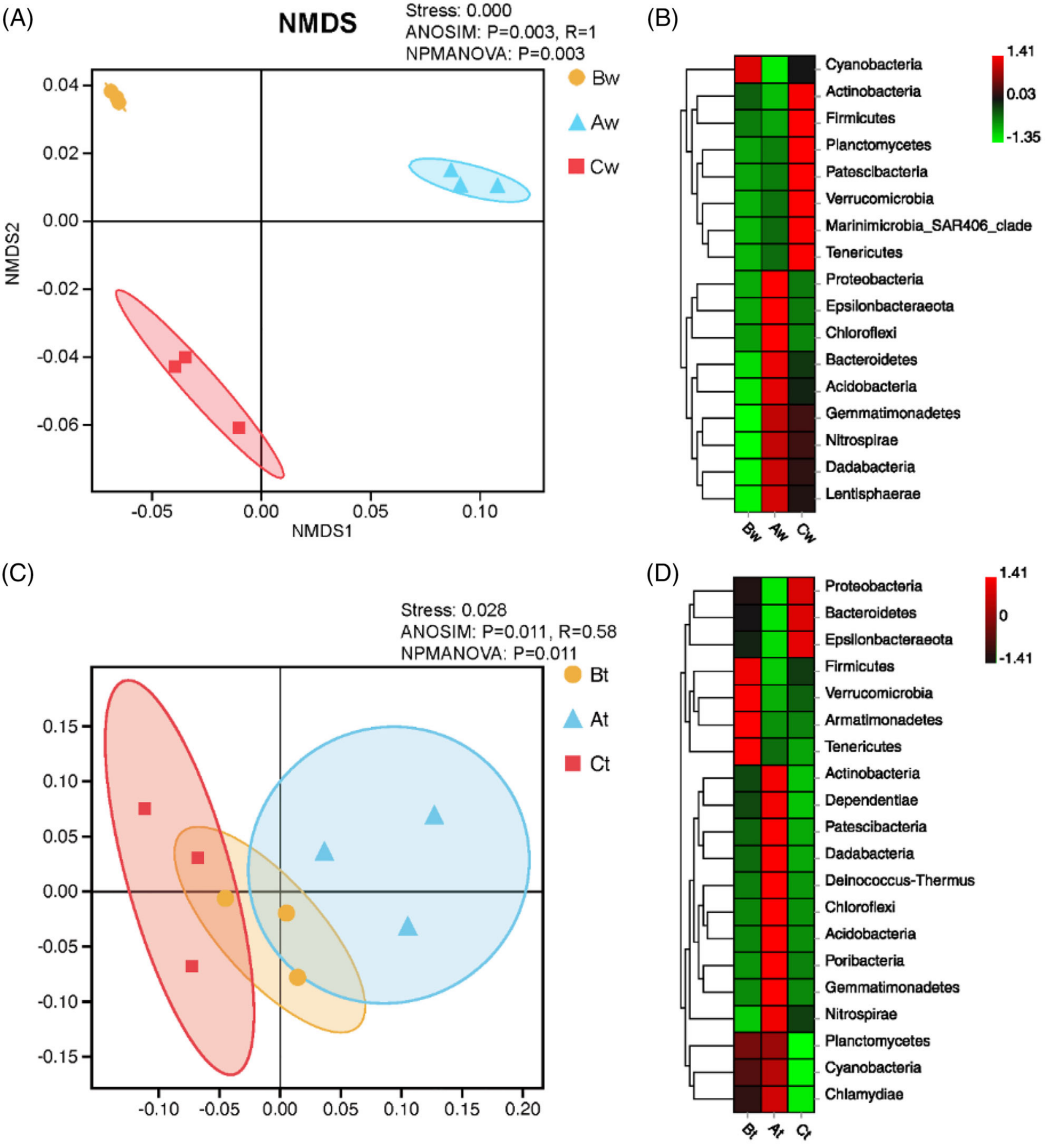
Beta diversity analysis of single sampling compartment from the three sites. The NMDS is based on weighted unifrac distances between microbiome communities of single compartment (A and C). Heat maps of bacterium abundance clustering (B and D). The top 20 phylum sample clustering (vertical clustering), and different colour indicates the various relative abundance of the phylum between groups (red means great abundance).

For beta diversity, the non‐metric multidimensional scaling (NMDS) highlights marked differences in taxonomic composition among the seawater samples at different sites (Figure 5A, stress: 0.000; Anosim: *p* = 0.003, *R* = 1; Npmanova: *p* = 0.003). Cyanobacteria was significantly higher in Site B; Proteobacteria, Epsilonbacteraeota, Chloroflexi, Bacteroidetes, Acidobacteria, Gemmatimonadetes, Nitrospirae, Dadabacteria and Lentisphaerae are higher in Site A; Actinobacteria, Firmicutes, Planctomycetes, Patescibacteria, verrucomicrobia, Marinimicrobia, Tenericutes are higher in Site C (Figure 5B). The NMDS also highlights significant differences in taxonomic composition among the tissue samples at different sites, however, less than the differences detected in water samples (Figure 5C, stress: 0.028; Anosim: *p* = 0.028, *R* = 0.58; Npmanova: *p* = 0.011). Firmicutes, verrucomicrobia, Armatimonadetes, Tenericutes are significantly higher in Site B; Acidobacteria, Dependentiae, Patescibacteria, Dadabacteria, Deinococcus, Chloroflexi, Actinobacteia, Poribacteria, Gemmatimonadetes, Nitrospirae are higher in Site A; Proteobacteria, Bacteroidetes and Epsilonbacteraeota are higher in Site C (Figure 5D). There are no significant differences in taxonomic composition among the sediment samples at different sites based on NMDS analysis (stress: 0.000; Anosim: *p* = 0.074, *R* = 0.5; Npmanova: *p* = 0.2988).

**FIGURE 6:**
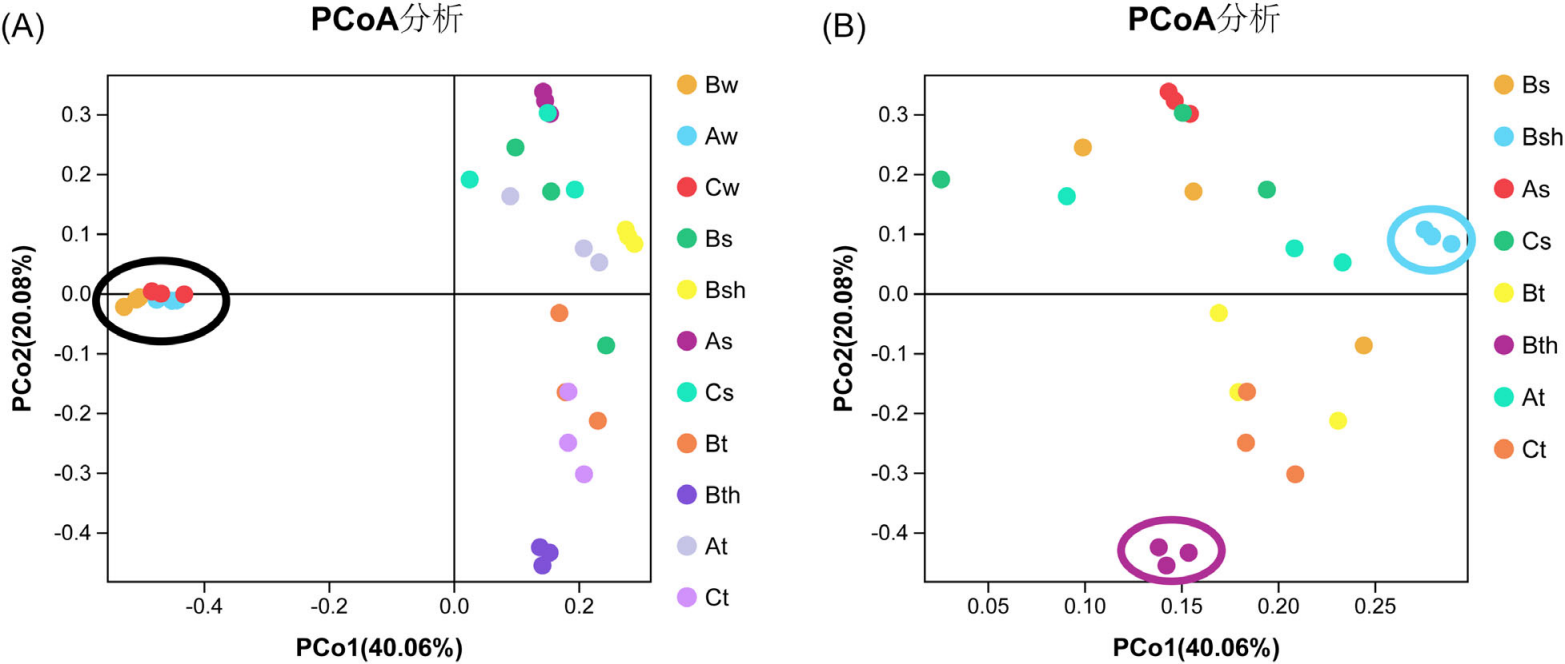
Clustering of water, sediment and coral tissue samples based on bacterial abundances on the family level. Ordination is based on a Bray–Curtis dissimilarity matrix and Pearson correlation in a principal coordinate analysis. Percentages signify the variance amount elucidated by each dimension. (A) Shows water, sediment, and tissue samples from all sites and indicates clustering of water samples from the three sites (in black circle). (B) Shows sediment and tissue samples after excluding water samples and indicate separation of healthy tissue from Site B (Bht) (in purple circle) and sediment near healthy colony from Site B (Bhs) (in blue circle)

To additionally detect variances in bacterial community structure across samples from different compartments, we plotted a principal coordinate analysis (PCoA) based on a Bray–Curtis dissimilarity matrix. Analysis of molecular variance (AMOVA) displayed that seawater samples were tightly clustered together but visibly detached from all coral tissue and sediment samples (Figure 6A). To further test whether differences between coral tissues and sediments occur, we left out seawater samples and accomplished a PERMANOVA across these two sets of samples (Figure 6B). Low differences were detected between coral tissues and sediments groups (Figure 6B). Coral samples were generally clustered except for the healthy colony Bht which was shown to be separate, and the same case applied to Bhs.

### Relationship between microbial diversity and water complexity

**FIGURE 7:**
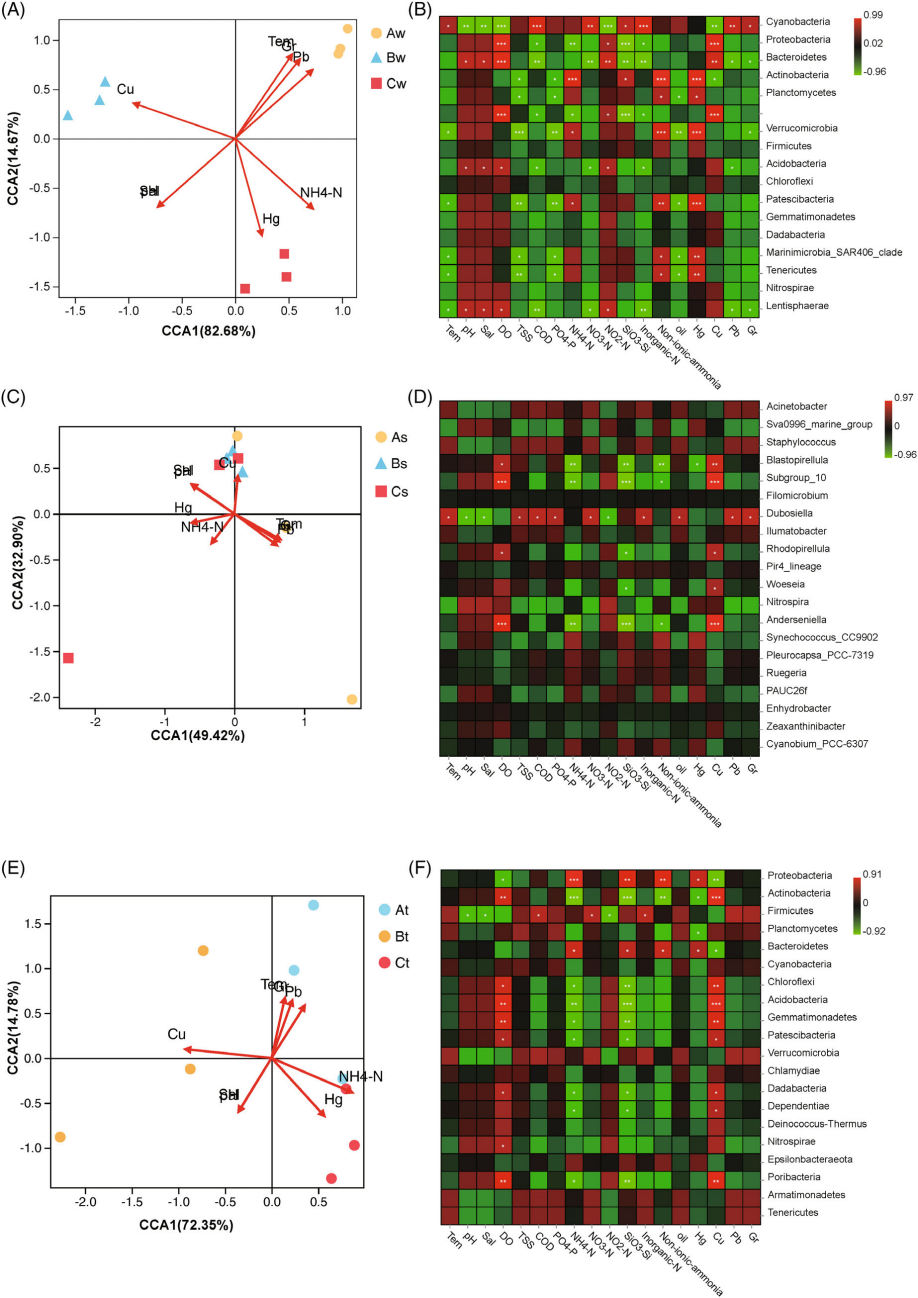
Canonical correspondence analysis (CCA) of the correlation between environmental variables and the bacterial community is represented in first element (CCA1) and second element (CCA2) for seawater (A, B), sediment (C, D), and tissue (E, F) respectively of the three selected sites, based on OTUs derived from SILVA dataset

To additionally explore variations in bacterial community structures across samples in relation to water complexity, the CCA ordination created from the OTUs obtained data was plotted as a two‐dimensional diagram (Figure 7). The water complexity is shown in table S1. The relationship between OTUs and the environmental limitations were represented by the first element (CCA1) and second element (CCA2), explained as 82.68% and 14.67% for seawater samples; as 49.42% and 32.90% for sediment samples; as 72.35% and 14.78% for coral tissue samples (Figure 7). Microbial community belongs to Site B seawater samples was positively associated with temperature (Bednarz et al., 2015), chromium (Gr) and plumbum (Pb); that belongs to Site A seawater samples was positively associated with cuprum (Cu); that belongs to Site C seawater samples was positively associated with mercury (Hg) and NH_4_‐H. In general, pH, salinity, and dissolved oxygen (DO) had the greatest influence on seawater microbial species composition. Cyanobacteria was mostly affected by chemical oxygen demand and inorganic‐N, while Proteobacteria and Rhodopirellula were mostly affected by CU and DO. Hg had the highest impact on Planctomycetes and together with non‐ionic ammonia had the highest impact on Synechoccus, while Rugeria relative abundance was mostly impacted by SiO_3_—Si and NH_4_—N (Figure 7A,B). Bacterial community associated with sediment was mostly affected by Cu and DO (Figure 7C,D).

Bacterial community associated with coral tissue from Site B was positively correlated with temperature, Pb, Gr and NH_4_—N. While that from Site A is correlated with Cu, Pb and salinity, and that from Site C is correlated with NH_4_—N and HG (Figure 7E). In general, DO and Cu had the greatest effect on the bacterial composition associated with coral tissue samples from the three sites. Proteobacteria and Bacteroidetes were mostly affected by NH_4_—N, SiO_3_—Si, non‐ionic ammonia and Hg (Figure 7E,F).

## DISCUSSION

Fast responding markers to coral ecosystem disturbance are required to develop an appropriate restoration plan. Here, we performed a range of statistical analysis approaches across host‐associated and multiple free‐living reef microbiomes in order to detect their fundamental value as sensitive indicators of environmental perpetuation. The exploration of coral reef microbial structure as a diagnostic tool for environmental fluctuations will enhance the capability of introducing of microbial monitoring in restoration of coral ecosystem.

### Coral reef microbiomes show compartment specificity

Compartment specificity of coral reef microbiome was investigated through comparison of the of microbial community similarities in sediment, water column, and *Porites lutea* tissue collected from three sites. Results indicated that water samples presented an overall lower diversity compared to sediment samples. This is credited to many factors including the dynamic nature of a water in contrast to the collective nature of sediment, the long sedimentation progression of particulate matter over decades as well as the tendency of microorganisms to attract to fine particles in the water column which later to be deposited into the sediment with loads of organic substances and hence will have higher susceptibility towards microbial diversity. In addition, the excessive number of cells usually found associated with sediment, also enhance the presence of high microbial diversity attributed to organic matter degradation (Lloyd et al., 2013). Since Cyanobacteria, primary producing phototrophs, are expected to boom in water columns during summer period because of high temperatures and extended light exposure, they dominated water samples together with Proteobacteria, with different reabundance among different sites, while in sediment, Proteobacteria, Actinobacteria and Planctomycetes were the most dominant. Each compartment shared the major 10 observed phyla with close abundance of most of the phyla but with specific differences in few phyla site wise. The Phylum Chloroflexi was shown to be abundant in Site C sediment compared to Sites A and B, which consists of various associates such as anaerobic organohalide respirers, filamentous anoxygenic phototrophs, and thermophiles, (Krzmarzick et al., 2012). Nevertheless, the abundant occurrence in Site C with the best coral health status suggests that they might have a key role in the biogeochemistry in this specific site. In tissue samples, Site A showed the highest abundance of Firmicutes, followed by Site C then Site B.

Remarkably, the most dominant bacteria in all sites were designated as core bacteria. Ten of the definite bacterial taxa that were enhanced in various clusters recognized in LEfSe analysis were core microbiome bacteria, including Pseudomonas (OTU1400 and OTU543), and Synechococcus_CC9902 (OTU274). This core holobiont are usually associated with enhanced environmental resilience and ecological role of the holobiont (Brener‐Raffalli et al., 2018; Hernandez‐Agreda et al., 2017).

Although tissues from all sites were dominant in Proteobacteria with Site C having the highest dominance followed by Sites A then B, interestingly, bacterial taxon Bacteroidia, represented in genus *Chlorobium* showed unique prominent appearance and significant abundance in the coral tissue replicates from healthy colony collected from severely impacted Site B (Bht) compared to unhealthy colonies collected from the same site. This points towards functional role of this taxon in coral host tissue and coral‐specific resistant microbiome as Bacteroidetes are reported to possess the mechanism for adaptation to live attached to substances, have the ability to breakdown polymers, such as some glycoside, peptidases, hydrolases, adhesion proteins, glycosyl transferases in addition to the genes for gliding motility (Fernández‐Gómez et al., 2013). This hypothesis needs further systemic analysis of the Bht colony for more confirmation.

NMDS detected an obvious separation of the microbial communities from different reef compartments (Figure 4), and compartment‐specificity was analysed with PERMANOVA. Furthermore, alpha diversities ANOVA varied significantly among reef compartments. Sediment harboured the highest diversity of bacterial community, followed by water then coral. This result suggests high specificity of coral microbiome to reef habitat. Our results are in consistence with previous reports by Carlos et al. (2013), Forest et al., 2002, Glasl et al., 2018, Tout et al., 2014, and Webster and Thomas, 2016. High compositional variations among different compartment microbiome could be due to interference from existent biotic factor or different nutrient availability.

### Variable against similar community assembly patterns

Microbial community assembly patterns were analysed for their uniformity against variability using Bray–Curtis similarity index (0 = dissimilar, 1 = identical) for samples collected from the same site. Results revealed a compositional similarity among tissue and sediment samples within the same site rather than among sites. On the other hand, water samples showed variable composition from tissue and sediment within the same site. The similar response of sediment and tissue microbiome to the unique environment of a specific site reflects linkage between microbial assembly in sediment and tissue from the same location as well as their similar response to the environmental change. However, water indicates unique microbial pattern, which suggests different functional response to the environmental conditions. These results indicate deterministic functional and geographical specificity drive the community patterns, which highlights the diagnostic value of these microbiomes as indicators of environmental variables. Microbiomes compositional similarity was further tested in water, sediment, and tissues among all three sampling Sites A, B and C. NMDS showed that the microbial community structure of water samples varied significantly among sites, though less significance was detected among tissue and even less among sediment from different sites. The high spatial variability of water microbiomes reflects that environmental perpetuation rather than habitat characteristics are the key factors shaping community pattern. These results suggest more functionality of water microbiome towards adaptation to environmental conditions than those of sediment and tissue. Coral‐ microbiomes showed slight change with the environmental conditions, revealing variable community pattern even inside the same species (Wiedenmann et al., 2013). A greater variation in community structure can cause raised community heterogeneity, as a common feature of host‐associated microbiomes (Casey et al., 2015; Glasl et al., 2016; Zaneveld et al., 2016).

Although the overall number of phyla revealed in the study sites varied among the stations/compartments, the most dominant and modest dominant phyla were disseminated at all the stations. The number of phyla represented here is analogous to results reported earlier in different marine environments, such as Pacific Ocean and the (SCS) (Walsh et al., 2016; Wang et al., 2018), as up to 47 phyla could be detected in sediments of the South China Sea (Wang et al., 2018) using NGS technology.

### Microbial response to environmental disturbance

Yet core bacterial microbiomes is known for high specificity (Hernandez‐Agreda et al., 2017); therefore, alterations in their abundance speculate the adaptive approach of coral holobionts according to ecological disturbance in various environments. Here, the modification of the associated bacteria in coral tissue among different sites was more noticeable than that in sediment and seawater, even though the key aspects affecting the local variation of the symbiotic bacteria were DO and Cu. The relative abundance of core bacterial microbiome in tissues isolated from Site C, shifted from high abundance *of Enhydrobacter* and *Psuedomonas* to *Sva 0996_marine group* in Site A with moderate coral health status signifying that the members of the core microbiome change from being dominant to rare bacterial species in the community structure under environmental stress (Li et al., 2014). The shift from abundance to scarcity detected in our study might be due to different environmental disturbances in Weizhou Island. The refuge theory hypotheses that SST rise caused by climate change will positively affect the growth of corals in the high‐latitude waters. This situation should theoretically apply to the coral reefs of Weizhou Island. However, dramatic deterioration of the living coral cover and coral assemblage is continuously detected in the area. Continuing human activities, for example, sewage discharge, tourism, marine engineering, and so on, could be the reason for coral deterioration (Cao et al., 2014; Yu et al., 2019) and prevention of coral reef from benefiting by the refugee theory. Zhao et al. (Zhao et al., 2014) reported that human activities have greater contribution to coral decline than global warming in Luhuitou fringing reef (Hainan). We support the hypotheses that coral decline is likely to happen due to a combination of factors. Even within the single factor, there are multiple sub‐branching factors all related to combination of causal agents responsible for coral decline.

For example, increased water eutrophication is caused by high levels of [DIN: NH_4_ + N, NO_2_ − N, NO_3_ − N] and phosphate [PO_4_ − P] coming from sewage discharge, industrial wastewater, fertilizers, and pesticides, in addition to fish farming, and so on. This increased eutrophication is positively correlated with macroalgal expansion and negatively correlated with coral calcification and fertilization and cause more susceptibility of corals to microbial pathogens (Voss & Richardson, 2006). Although high level of DIN and DIP was long known for their adverse effect towards coral health, they have been also reported to increase growth rate of zooxanthellae associated with corals with no direct negative effect detected on coral growth (Huang et al., 2019). So, both levels of nutrient pollution and the ratios of N:P can shape the influence of nutrients on coral health.

Moreover, coral reefs with high DIN become more susceptible to coral decline when exposed to high temperature, suggesting that high DIN is of a risk to coral when combined with high temperature, which weakens coral tolerance and adaptation skills towards temperature elevation. Presence here of some bacterial phylum such as sulfate‐reducing bacteria and Planctomycetes propose candidates that indicates contamination concentration. This result speculates that sediments at contaminated sites are inhabited by bacteria with a higher ability to diminish intracellular amounts of heavy metals, antibiotics, or other ecological pollutants.

As we can see from the Weizhou Island map (Figure 1), Site A is located in the south east of Weizhou Island and is the closest to the shoreline suggesting great impact with the human activity coming from sewage, industrial wastewater and other pollution. While Site C showed the least impacted site with the human activity and the best of living coral cover. This is in consistence with Chen et al. (Chen et al., 2021) who reported that erosion in the south of Weizhou Island was obviously greater than in the north since eutrophication in the south is more significant. However, Site B showed the least coral cover, this could be due to high sedimentation. Huang et al. (Huang et al., 2019) also revealed that developed tourism in the area lead to more sewage into the sea water. This results in more nutritional concentration and hence more algal pollution which in turn, over compete with coral growth. Thus, a broad diversity and combination of potential nutrient effects introduced through human activities will certainly be the keys to the understanding of the microbial shift associated with coral decline and hence to the development of successful coral rescuing strategies.

In summary, our results indicated high levels of bacterial community flexibility with remarkable variations among the three sites, whereas no noteworthy differences were detected in environmental parameters among these sites. Our results are in consistence with and support the previous report by Yu et al (Yu et al., 2019) who suggested that both decline in the coral assemblages and shift in the coral microbial community structure in Weizhou Island are more likely to be structured by the elevated human activities, rather than natural disturbances. For example, abiotic factors such as marine pollution caused by shipping, overfishing, petroleum factories in the area, uncontrolled tourism, all of which lead to biological factors, such as coral pathogens and algal expansion cause diseases due to shift in the richness, pattern, and dominance of coral‐symbiotic bacteria (Ceh et al., 2012; Cróquer et al., 2013; Littman et al., 2011; Morrow et al., 2015; Yu et al., 2021).

## CONCLUSIONS

Water showed the most prominent habitat with significant markers detected at different sites, followed by tissue microbiome, while sediment showed no significant difference among sites. Our conclusion is based on (1) reef compartment‐specificity, (2) similarity and variability of its community assembly, and (3) sensitivity to environmental variability. The flexibility in dominance/abundance of coral symbiotic bacteria as well as water and sediment associated bacteria is a significant basis for the ecological acclimatization of corals, which might promote the coral resilience and tolerance around Weizhou Island. The different reef compartments exhibited different core microbiome patterns; nevertheless, there is still a group of bacteria that are mutual and independent of the ecological alterations among locations, thus, offering proof for a symbiosis that can adapt to dissimilar environmental surroundings. Site A with moderately damaged coral reef status showed more stable bacterial dominance over different locations, which is in consistence with previous results from studies on tropical and subtropical Taiwan over 2 years (Yang et al., 2017) compared to Site C which present mostly healthy coral site, suggesting that damage of coral reef in Site A might be temporary and will vary over different seasons, and more investigations need to be added for confirmation.

Our results propose a correlation between environmental disturbances and the coral reef sediment, water and tissue microbiome and offer a vision into the role of microbial community shift in adapting the disturbing environment. The flexibility and stability of the coral‐bacterial composition reveal that corals can acclimatize to ocean acidification and other environmental stress by regulating their holobiont; drawing attention to their importance as a significant tool in coral restoration through close identification of the shift in microbial bacterial association. We proposed that the great diversity and abundance of coral‐associated bacteria and the increase in *Chlorobium* abundance in tissue of coral *Porites lutea* can help the host retain its physiological functions and the negative physiological effects of the decrease in density under certain environmental stress.

## CONFLICT OF INTEREST

The authors declare that there is no conflict of interest.

## Supporting information


**Figure S1** **Relative abundance of major 10 bacterial Phylum, Order, and Genus groups based on OTUs derived from the SSU rDNA of different sampling.** Each colour denotes one of the 11 most abundant Phylum, Order, and Genus (overall sequence count) in all samples. The rest of other taxa are indicated under group “others”. BW, AW, and CW represent water sample from site B, A, and C sites respectively. Bs, Bhs, As, Cs represent sediment samples from B, near healthy colony collected from site B, A, and C site respectively.

**Table S1** Supporting information

**Table S2** Supporting information

## Data Availability

All data sequences have been deposited to the NCBI website and is available to the public.
