# Supplementary figures and images for: The bacterial signature offers vision into the machinery of coral fitness across high‐latitude coral reef in the South China Sea
Source: Environ Microbiol Rep. 2022 Aug 31;15(1):13–30. doi: 10.1111/1758-2229.13119 (PMC10103774; doi:10.1111/1758-2229.13119)

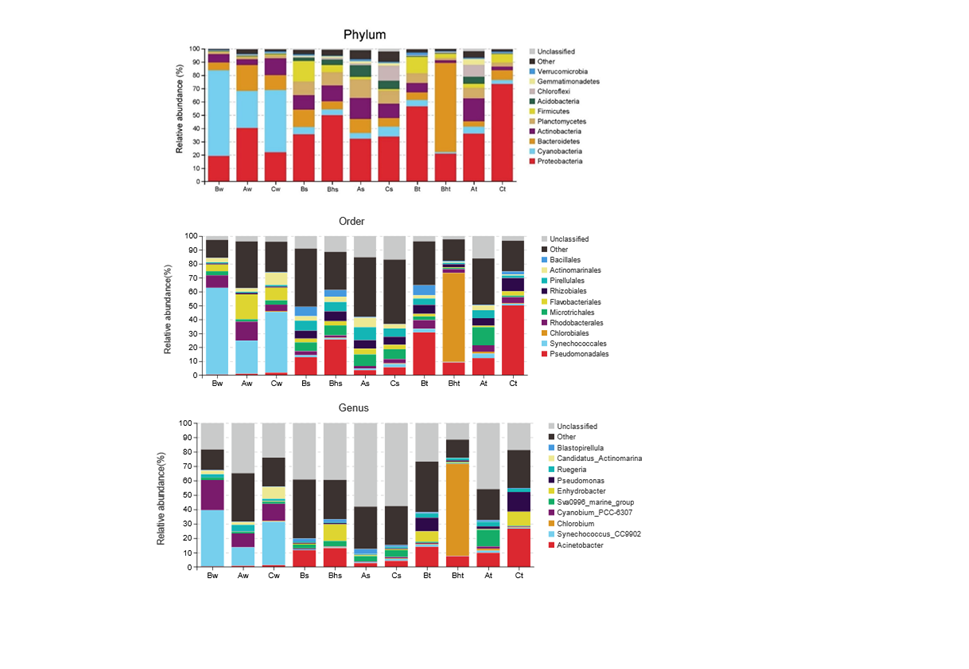

Supplement: Supplementary file 1 — Figure S1 Relative abundance of major 10 bacterial Phylum, Order, and Genus groups based on OTUs derived from the SSU rDNA of different sampling. Each colour denotes one of the 11 most abundant Phylum, Order, and Genus (overall sequence count) in all samples. The rest of other taxa are indicated under group “others”. BW, AW, and CW represent water sample from site B, A, and C sites respectively. Bs, Bhs, As, Cs represent sediment samples from B, near healthy colony collected from site B, A, and C site respectively. [file EMI4-15-13-s002.png]
